# Postmenopausal hyperandrogenism due to an ovarian sex cord-stromal tumour causing elevated dehydroepiandrosterone sulphate: a case report

**DOI:** 10.1186/s12905-022-01879-8

**Published:** 2022-07-17

**Authors:** Manilka Sumanatilleke, Nipun Lakshitha de Silva, Gayani Ranaweera, Chinthaka Appuhamy, Kanishka Karunaratne, M. V. Chandu de Silva

**Affiliations:** 1 Diabetes and Endocrine Unit, National Hospital of Sri Lanka, Colombo, Sri Lanka; 2 Department of Clinical Sciences, Faculty of Medicine, General Sir John Kotelawala Defence University, Colombo, Sri Lanka; 3 Department of Pathology, Faculty of Medicine, University of Colombo, Colombo, Sri Lanka; 4 Department of Radiology, North Colombo Teaching Hospital, Colombo, Sri Lanka; 5 Department of Obstetrics and Gynaecology, Faculty of Medicine, University of Colombo, Colombo, Sri Lanka

**Keywords:** Hyperandrogenism, DHEAS, Dehydroepiandrosterone sulphate, Ovary, Sex cord-stromal tumour

## Abstract

**Background:**

The source of excess androgen can be obscure in postmenopausal women with new-onset hyperandrogenism. If serum dehydroepiandrosterone sulphate (DHEAS) is raised, it is presumed to be of adrenal origin because DHEAS is exclusively produced from adrenal cortical cells. This reports an elderly female presenting with new-onset hyperandrogenism due to an ovarian sex cord-stromal tumour, associated with increased serum DHEAS levels.

**Case description:**

A 76-year-old female with long-standing diabetes and hypertension presented with hirsutism and male type alopecia for six months. She had menopause at 55 years of age. There was a pelvic mass on examination. Total testosterone was 6.106 ng/ml (0.124–0.357) and DHEAS was > 1000 µg/dL (35–430). Contrast-enhanced computed tomography of the abdomen and pelvis showed a heterogeneously enhancing complex mass measuring 11 × 8 cm in the left adnexal region. Adrenal glands were normal. She underwent total abdominal hysterectomy, bilateral salphingo-oophorectomy, and omentectomy. Both testosterone and DHEAS normalised following surgery. Histology revealed a sex cord-stromal tumour, likely a steroid cell tumour with malignant potential. Fluorodeoxyglucose-Positron emission tomography did not show any additional lesions.

**Conclusions:**

Due to the lack of sulfotransferase in ovarian tissue, markedly elevated DHEAS originating from an ovarian neoplasm is unusual. This phenomenon has not been described except in a patient with a steroid cell tumour causing Cushing syndrome and hyperandrogenism. The mechanism of this rare occurrence remains elusive. Knowledge of this unusual presentation would enable the clinicians to be cautious in localising the androgen source in women with hyperandrogenism.

## Background

New-onset hyperandrogenism is extremely rare in postmenopausal women compared to that of premenopausal women. Common causes in postmenopausal women include androgen-secreting tumours of the ovary or adrenal and ovarian hyperthecosis [1, 2]. Types of ovarian tumours associated with hyperandrogenism include Leydig cell tumours, Sertoli cell tumours, and steroid cell tumours [3].

Differentiation of the cause of hyperandrogenism is mandatory in these women to recognise underlying endocrine disorders. In a postmenopausal woman with new-onset hyperandrogenism, imaging of the adrenals and ovaries guides the localisation of the source of androgen excess. However, small ovarian tumours producing androgens can be missed during imaging. Adrenal incidentalomas can further complicate the diagnostic workup [2]. Though combined adrenal and ovarian venous sampling can be helpful in such a scenario, it is technically demanding and lacks standardisation [4]. Dehydroepiandrosterone sulphate (DHEAS) is considered to be exclusively produced from the adrenal glands. Therefore, markedly elevated DHEAS in a patient with hyperandrogenism is indicative of an adrenal source. There are no reported cases of markedly elevated serum DHEAS levels in postmenopausal women with hyperandrogenism due to ovarian tumours.

This report describes a postmenopausal woman with an ovarian sex cord-stromal tumour causing new-onset hyperandrogenism associated with markedly elevated serum DHEAS.

## Case presentation

A 76-year-old female presented with new-onset gradually worsening hirsutism over the face, upper chest, upper abdomen, and arms and frontal balding for six months. She had not noticed acne, clitoromegaly, or changing voice. There was no abdominal pain, bloating, bowel or urinary symptoms. She had attained puberty at 13 years and had regular menstruation up to 55 years. Her two childbirths were uncomplicated. She had not experienced postmenopausal bleeding or hot flushes. There was no exposure to exogenous androgenic substances.

Past medical history included satisfactorily controlled diabetes and hypertension for 15 years and right total hip replacement for osteoarthritis 10 years ago. There was no history of microvascular or macrovascular complications of diabetes.

On examination, she was of average built and looking well. She had thick terminal hair over her face, upper chest, upper abdomen, and arms and there was no clitoromegaly. Abdominal examination revealed a firm pelvic mass up to the level of the umbilicus. The examination was otherwise normal.

Total testosterone and serum DHEAS were markedly elevated and confirmed on repeat testing. Serum DHEAS was performed using the chemiluminescence method on the IMMULITE platform. The results of her blood investigations are summarised in Table 1.

**Table 1:** Summary of investigations at the time of presentation

Investigation	Result	Reference range
Haemoglobin (g/dL)	15.5	11–16
Mean corpuscular volume (fl)	89.6	80–96
White cell count (× 10^9^/L)	7.8	4–11
Platelet count (× 10^9^/L)	242	150–450
Urinalysis	Normal	
Serum creatinine (mg/dL)	0.82	0.7–1.2
Serum sodium (mmol/L)	135	135–145
Serum potassium (mmol/L)	4.8	3.5–5.1
HbA1c	7.8%	< 5.7%
Thyroid-stimulating hormone (mIU/l)	4.56	0.5–4.7
Low dose dexamethasone suppression test (nmol/L)	25	< 50
Total testosterone (ng/mL)	6.106	0.124–0.357
Dehydroepiandrosterone sulphate (µg/dL)	> 1000	35–430

**Fig. 1:**
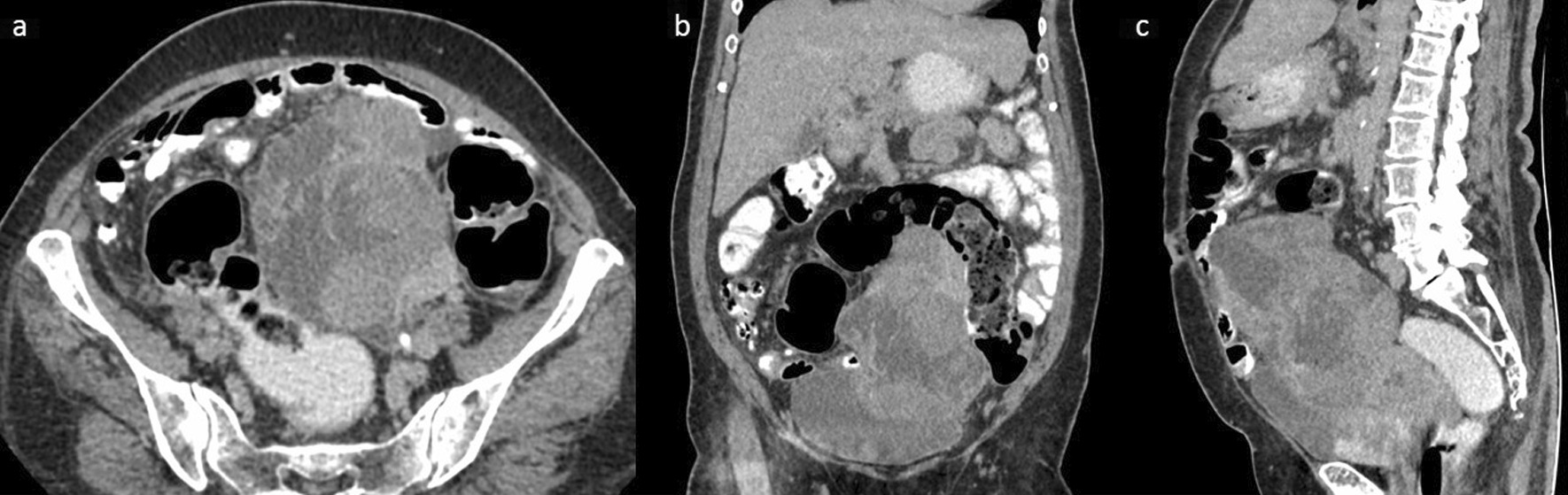
Contrast-enhanced CT of the abdomen and pelvis showing a heterogeneously enhancing complex mass arising from the left adnexal region. In the axial image (**a**), it measures 11 × 8 cm. There is no calcification, fat content, or air-fluid level within. In the coronal (**b**) and sagittal (**c**) images, it extends up to the level of the umbilicus abutting the anterior abdominal wall. Mass merges with uterus and bladder with no intervening fat planes. The mass displaces the small and large bowel

Contrast-enhanced computed tomography of the abdomen and pelvis revealed a heterogeneously enhancing complex mass measuring 11 × 8 cm in the left adnexal region. There was no calcification, fat content, or air-fluid level within (Fig. 1). Bilateral adrenal glands appeared normal. There were no focal lesions in the liver.

**Fig. 2:**
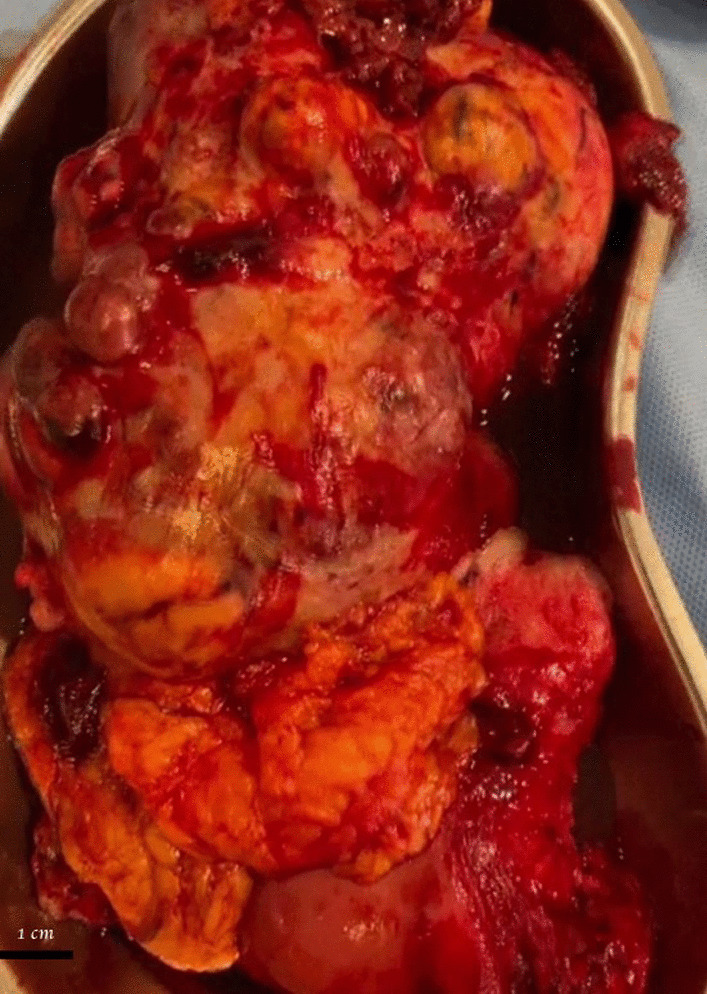
Gross appearance of the surgical specimen. Yellowish left ovarian tumour measuring 16 cm × 8 cm × 9 cm with haemorrhagic and necrotic areas

She underwent total abdominal hysterectomy, bilateral salphingo-oophorectomy, and omentectomy. A left ovarian tumour adherent to the lateral pelvic wall, measuring 16 cm × 8 cm × 9 cm showing a yellowish-brown cut surface with haemorrhagic and necrotic areas was removed completely (Fig. 2). The tumour was friable on removal. Pelvic or para-aortic nodes were not palpable. Uterus, right ovary, fallopian tubes, pouch of Douglas, liver, diaphragm, stomach, spleen, adrenals and lesser sac were macroscopically normal. Washing was not performed. Recovery was uneventful. One week after surgery, total testosterone dropped to 0.3 ng/ml (0.124–0.357) and serum DHEAS was 83 µg/dL. Whole-body Fluorodeoxyglucose-Positron emission testing (FDG-PET) scan after surgery did not show any additional FDG avid foci.

**Fig. 3:**
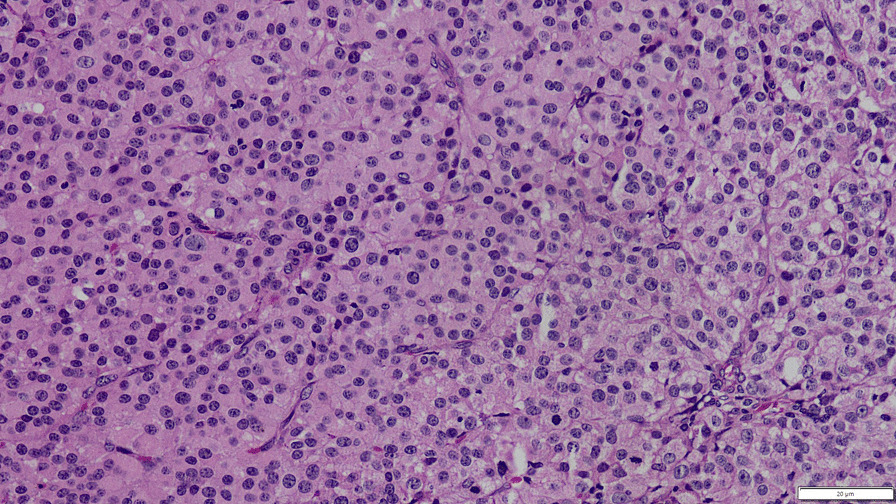
Microscopic appearance of the Haematoxylin and Eosin-stained surgical specimen in × 400 magnification. There are nests and sheets of polygonal cells with abundant eosinophilic cytoplasm and brisk mitotic activity. There is moderate nuclear pleomorphism. Occasional spindled cells are present

Figure 3 shows the microscopic appearance of the Haematoxylin and Eosin-stained surgical specimen in × 400 magnification (10 × 40 objective lenses) taken on an Olympus Bx43 microscope using an Olympus U-TV0.35XC-2 camera. The figure was acquired using the Olympus cellSens software with a resolution of 96 psi. Microscopically the tumour consisted of closely packed polygonal and occasional spindle-shaped cells with focal intervening vasculature. Cells were plump and focally contained moderately pleomorphic, enlarged nuclei with coarsely clumped chromatin. The cytoplasm was eosinophilic and abundant with occasional cells showing vacuolated cytoplasm. The reticulin stain highlighted reticulin fibres around the nests of cells. Capsular invasion, areas of necrosis, haemorrhage, and brisk mitotic activity (15/10 high power fields) were noted. Lymphovascular invasion was not detected. There were no tumour deposits on the ovarian surface. A nest of tumour cells suspicious of a deposit was seen on the right fallopian tube. Reinke crystals characteristic of Leydig cells were not seen in Haematoxylin and Eosin (H&E) and PAS (Periodic Acid-Schiff) stains. Pancytokeratin immunohistochemical stain showed strong and diffuse cytoplasmic staining of the tumour cells. There was intense cytoplasmic positivity with Calretinin, whereas the tumour cells were negative for inhibin stain. Histology was in keeping with a potentially malignant sex cord-stromal tumour of the left ovary. A steroid cell tumour showing potential malignant behaviour was the favoured diagnosis.

The patient showed improvement of alopecia and hirsutism three months after surgery. Considering the malignant potential of the tumour, options of chemotherapy and pelvic radiotherapy were discussed with the patient and she opted for radiotherapy.

## Discussion

Normal adrenal imaging and complete normalisation of serum DHEAS following surgery in our patient confirm that marked elevation of DHEAS was due to the androgen-secreting tumour of the ovary. Measurement of DHEAS using immunoassays is known to be accurate with minimal effect of assay interferences due to very high serum concentrations compared to other androgens and the presence of highly specific antibodies [5, 6].

Marked elevation of serum DHEAS is characteristically seen in patients with an adrenal origin of hyperandrogenism. An adrenal tumour must be strongly considered in a postmenopausal woman with DHEAS > 700 µg/dL [7].

**Fig. 4:**
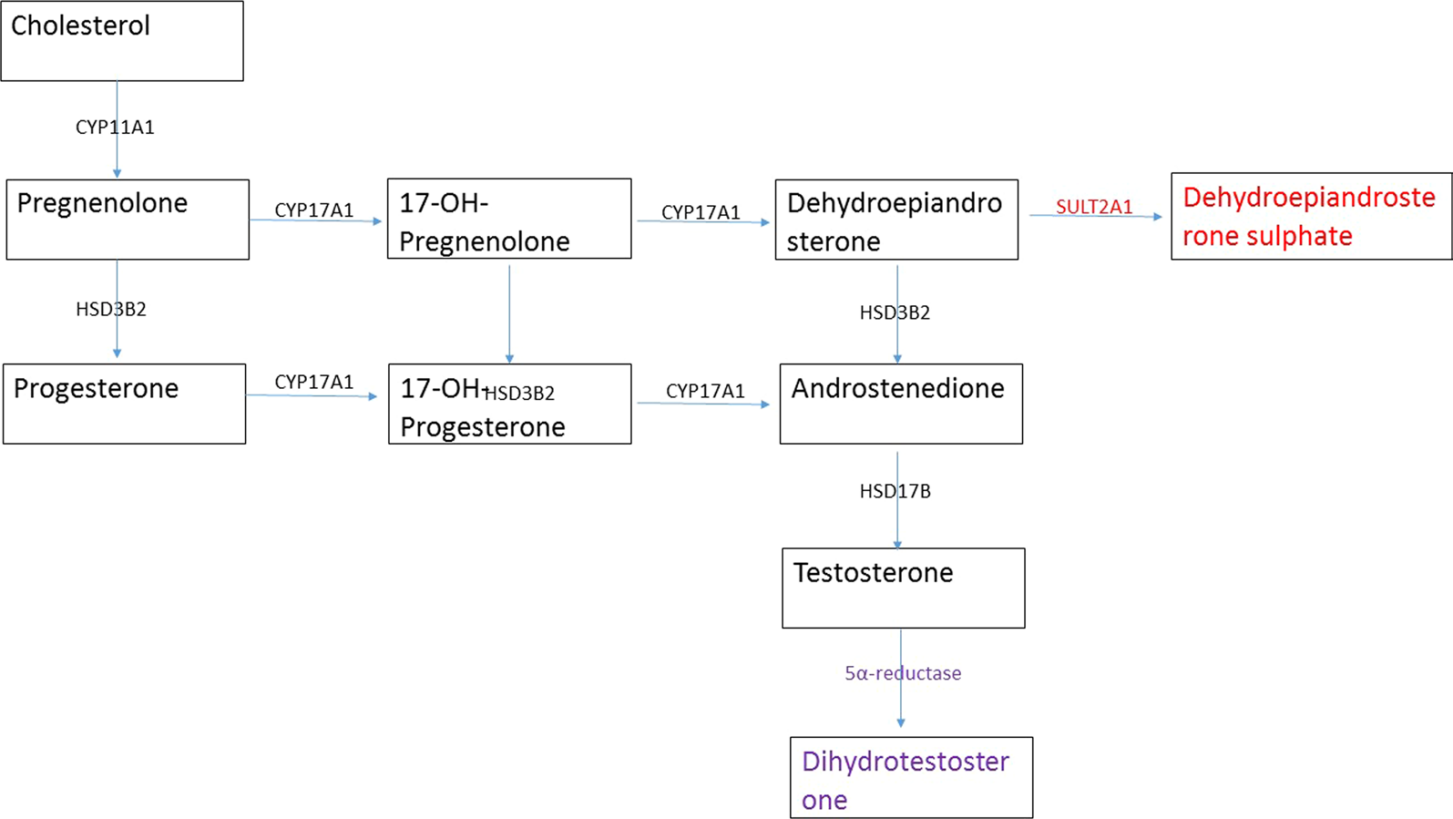
Steps of androgen synthesis. Synthesis of dehydroepiandrosterone (DHEA), androstenedione, and testosterone are common to both adrenal and ovary. Conversion of DHEA to dehydroepiandrosterone sulphate (DHEAS) is specific to the adrenal cortex. Conversion of testosterone to dihydrotestosterone is seen in peripheral tissues. CYP11A—P450 side-chain cleavage enzyme, CYP17A1—17α hydroxylase, HSD3B2—3β hydroxysteroid dehydrogenase type 2, SULT2A1—sulfotransferase 2A1

Lack of elevation of serum DHEAS in a patient with an ovarian source of hyperandrogenism could be explained by the female androgen synthetic pathway. In a postmenopausal woman, both ovaries and adrenal glands would contribute to the androgen levels in the blood. In the descending order of serum concentration, the main androgens in a female include DHEAS, DHEA, androstenedione, testosterone, and dihydrotestosterone [8]. Figure 4 describes the major steps of androgen synthesis in the human ovary, adrenal, and peripheral tissue. Testosterone, Androstenedione, and DHEA are produced from both ovaries and the adrenal cortex. However, DHEAS is produced by converting dehydroepiandrosterone (DHEA) by the enzyme encoded by the gene sulfotransferase 2A1 (SULT2A1). This enzyme is only present in the cells of the zona reticularis of the adrenal gland. Therefore, the adrenal gland is the only source of DHEAS. Studies in women undergoing oophorectomy have shown that DHEAS levels do not change significantly after the surgery, suggesting that ovaries do not contribute to serum DHEAS concentration [9]. Dehydroepiandrosterone from other tissues and exogenous sources can undergo sulphation to DHEAS at the adrenal gland [10]. DHEA levels are present at much lower concentrations in patients with ovarian tumours and exogenous use so that DHEAS elevation due to adrenal conversion would not be so marked [5]. In addition, in the reported literature describing elevated DHEA due to ovarian tumours, there are no cases of markedly elevated DHEAS. Therefore, adrenal conversion of ovarian DHEA is unlikely in our patient though we have not tested her DHEA levels.

As described earlier, SULT2A1 is only expressed in adrenal zona reticularis cells. However, there are cases of paraendocrine manifestations such as Cushing syndrome, hypercalcaemia, and erythrocytosis in patients with steroid cell tumours of the ovary [11–13]. These tumours can contain cells histologically resembling adrenocortical cells [14], probably explaining adrenocorticotropic hormone-independent Cushing syndrome in these patients. Some hypothesise that these tumours might have precursor cells originating from the adrenal cortex [12]. Other explanations would be the differentiation of tumour cells towards adrenocortical cells or the expression of unusual proteins in the tumour cells due to mutations. In a patient with Cushing syndrome due to a steroid cell tumour, DHEAS had been elevated [12]. Therefore, increased DHEAS in our patient could be explained through a similar mechanism. However, she did not have evidence of Cushing syndrome.

Ovarian neoplasms producing postmenopausal hyperandrogenism are rare compared to all the types of ovarian neoplasms. Sex-cord stromal tumours, including steroid cell tumours, Leydig cell tumours, Sertoli cell tumours, and Sertoli-Leydig cell tumours, are known to produce androgens [1]. Compared to all types of ovarian tumours, these are rare and tend to occur mostly in younger women [15]. It was difficult to ascertain the exact histopathological type out of the above possible types of sex-cord stromal cell tumours in our patient, though steroid cell tumour was the most likley. It would have been interesting to perform further immunochemical staining to see whether the conversion of DHEA to DHEAS took place in our patient’s tumour cells. This would allow further characterisation of these tumour types in the future.

In addition, the key lesson that must be taken from this patient’s history is that localising the source of hyperandrogenism in a postmenopausal woman has to be performed carefully. While it is well known that clinical presentation, imaging, or catheter studies have their limitations, DHEAS, which is considered to be a strong indicator of adrenal androgen excess, also can mislead the clinician.

## Conclusions

This reports the presentation of new-onset postmenopausal hyperandrogenism due to a sex-cord stromal tumour, unexpectedly associated with markedly elevated serum DHEAS levels. Further studies to understand its pathophysiological mechanism are warranted. Clinicians should play extreme caution in utilising available modalities in localising the source of excess androgen in postmenopausal women.

## Data Availability

The authors declare that data supporting the findings of this case report are available within the article, including Table 1 and Figs. 1, 2 and 3.

## Abbreviations

DHEA: Dehydroepiandrosterone
DHEAS: Dehydroepiandrosterone sulphate
